# Necrotizing enterocolitis in a term newborn after spontaneous cerebral parenchymal hemorrhage: a case report

**DOI:** 10.1186/s12887-024-04866-0

**Published:** 2024-06-08

**Authors:** Lijuan Zhang, Weifeng Lu

**Affiliations:** Department of Surgical Intensive Care Unit, Children’s Hospital of Nanjing Medical University, No.72 Guangzhou Road, Nanjing, Jiangsu Province 210008 China

**Keywords:** Necrotizing enterocolitis, Spontaneous cerebral parenchymal hemorrhage, Term newborn, Brain-gut axis

## Abstract

**Background:**

Necrotizing enterocolitis (NEC) and intracranial hemorrhage are severe emergencies in the neonatal period. The two do not appear to be correlated. However, our report suggests that parenchymal brain hemorrhage in full-term newborns may put patients at risk for NEC by altering intestinal function through the brain-gut axis.

**Case presentation:**

We present a case of spontaneous parenchymal cerebral hemorrhage in a full-term newborn who developed early-stage NEC on Day 15.

**Conclusions:**

It is possible to consider brain parenchymal hemorrhage as a risk factor for the appearance of NEC. Clinicians should be highly cautious about NEC in infants who have experienced parenchymal hemorrhage. This article is the first to discuss the relationship between parenchymal hemorrhage and NEC in full-term newborns.

## Background

NEC is the most common gastrointestinal emergency in neonatal intensive care units (NICUs) [1]. This disease mainly affects preterm infants, especially those with a low line birth weight (< 1500 g) [2]. However, fewer than 10% of NEC cases occur in full-term newborns [3]. NEC in full-term infants can be categorized into two subtypes: early-onset (≤ 7 days) and late-onset (> 7 days), with early-onset being more prevalent and late-onset associated with higher mortality rates [3–5]. The exact etiology and pathogenesis of NEC are not fully understood. Prematurity, low birth weight, enteral feeding, infections, gastrointestinal immaturity, intestinal hypoxia-ischemia, and microbial dysbiosis contribute to inducing an uncontrolled inflammatory response in the gut and lead to the development of NEC [6, 7]. Furthermore, some authors have proposed that disruptions in the gut-brain axis may also play a role in the pathogenesis of NEC [8]. There are relatively few reports of NEC in term infants, and most are associated with clinical factors such as perinatal asphyxia, sepsis, cyanotic heart disease, or endocrine disruption [4].

Spontaneous parenchymal hemorrhage is extremely rare in full-term newborns, and most have no apparent etiology or predisposing factors due in part to the rupture of an intracranial aneurysm [9]. The most common symptom is seizures, and some patients present with obvious signs of increased intracranial pressure, including full fontanelles, papilledema, and increased head circumference. In contrast, others present with nonspecific symptoms such as vomiting, apnea, or lethargy, which may result from increased intracranial pressure.

To our knowledge, NEC has not previously been reported in term newborns after spontaneous cerebral parenchymal hemorrhage.

## Case presentation

**Fig. 1:**
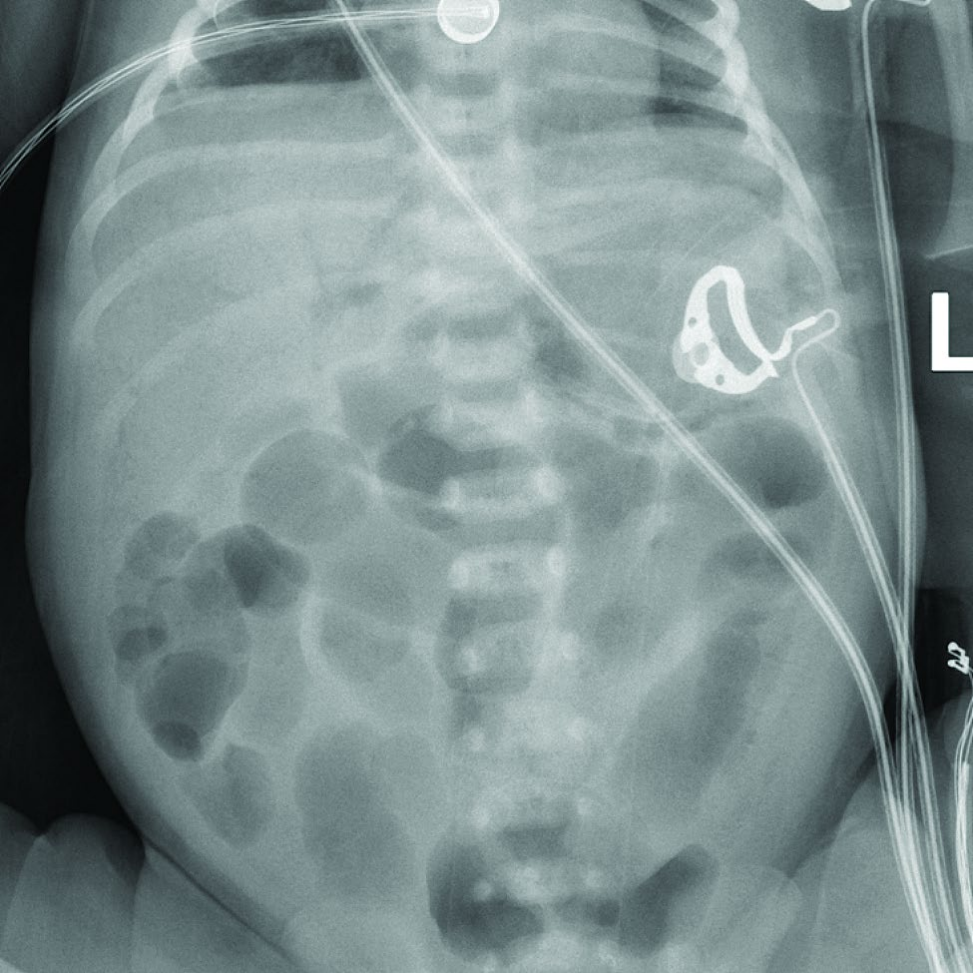
Plain abdominal X-ray (a) showed portal venous gas, bowel distension, widened inter-loop spaces, and focal pneumatosis intestinalis, without any free subdiaphragmatic air (pneumoperitoneum)

We described a 38-week full-term infant who was born vaginally, with Apgar scores of 10 at 1 and 5 min after birth. The newborn’s 3550 g birth weight, length, and head circumference were all within normal ranges for the gestational age. There was no history of trauma or recent infection. He was fed formula milk on the day of his birth, with a milk volume of 20–40 ml every 3 h. He received treatment for jaundice on the second day after birth and experienced recurrent convulsions during his hospitalization, characterized by shaking of the limbs. His cranial magnetic resonance imaging (MRI) revealed a heterogeneous signal hematoma in the right frontal lobe, subarachnoid hemorrhage, and ischemic foci around the lateral ventricles, and these lesions were considered possible cerebral vascular anomalies. He was transferred to our hospital’s care unit on the 5th day after birth; on examination, the patient was drowsy and lethargic with a tense anterior fontanelle and moderate jaundice of the skin all over the body. The remainder of the physical examination was ordinary. Moreover, the 33-year-old mother is in good health with no history of smoking, alcohol consumption, or drug use. Although she was diagnosed with gestational hypertension during this pregnancy, she did not require medication. The delivery was uncomplicated, with no occurrences such as premature membrane rupture, preeclampsia, or gestational diabetes. He was given sodium phenobarbital to control convulsions, mannitol to lower the intracranial pressure, cephalosporin to prevent infections, hemostatic agents, vitamin K1, calcium, and other treatments, and no apparent convulsions were seen during his hospitalization. The patient’s heart rate and blood pressure were monitored as usual, and oxygen saturation was maintained above 95% during nasal cannula oxygenation. At the time of admission, white blood cell (WBC) and c-reactive protein (CRP) were not significantly elevated, hematocrit (HCT) was 40.2%, total bilirubin was 14.4 mg/dl, unconjugated bilirubin was 13.0 mg/dl, and there were no significant abnormalities in electrolytes, blood glucose, stool routine, urinalysis, or cardiac ultrasound. During this period, formula milk (osmolality 317 mOsm/kg calorie 67 kcal/100 ml) was gradually increased at a rate of 10–20 ml/kg/day, according to the standardized feeding regimen of the neonatal intensive care unit. The jaundice subsided on postnatal Day 8, and a decrease in milk intake was observed on postnatal day nine at a feeding rate of approximately 110 ml/kg/day, at which point the abdominal ultrasound results were reassuring. A review of cranial MRI showed no significant progression of the intracranial hemorrhage. A change of formula with lower osmolality (high calorie deeply hydrolyzed formula osmolality 195 calories 80 kcal/100 ml) was given, decreased milk intake reappeared on Day 14 of the postnatal period, followed by refusal to feed, abdominal distension, bloody stools on Day 15 of the postnatal period, abdominal X-ray showed portal venous gas, bowel dilatation, widened inter-loop spaces, and focal pneumatosis intestinalis. Notably, no evidence of free intraperitoneal gas beneath the diaphragm is observed (Fig. 1), ultrasound showed pneumoperitoneum along with elevation of CRP, and he was subsequently diagnosed with NEC. The patient was treated with fasting, gastrointestinal decompression with the upgraded antibiotic meropenem, and total parenteral nutrition and enteral feeding were gradually reintroduced seven days after the onset of NEC without any disease recurrence. The patient was discharged 29 days after birth in good clinical condition.

## Discussion and conclusions

The patient was a term normal-weight infant with spontaneous parenchymal brain hemorrhage who presented with NEC on postnatal Day 15. Although early-onset NEC is more common in term infants, our case presented with typical symptoms on the 15th day after birth. The patient did not experience perinatal asphyxia, and tests during hospitalization including cardiac ultrasound, thyroid function, and genetic metabolic screening were all normal. Blood culture was negative, and there were no clear signs of infection based on WBC count, CRP, and procalcitonin levels. These factors are known susceptibilities for NEC in full-term infants according to existing literature [4], which reinforces our speculation that the spontaneous cerebral parenchymal hemorrhage in the patient is associated with the subsequent development of NEC.

**Fig. 2:**
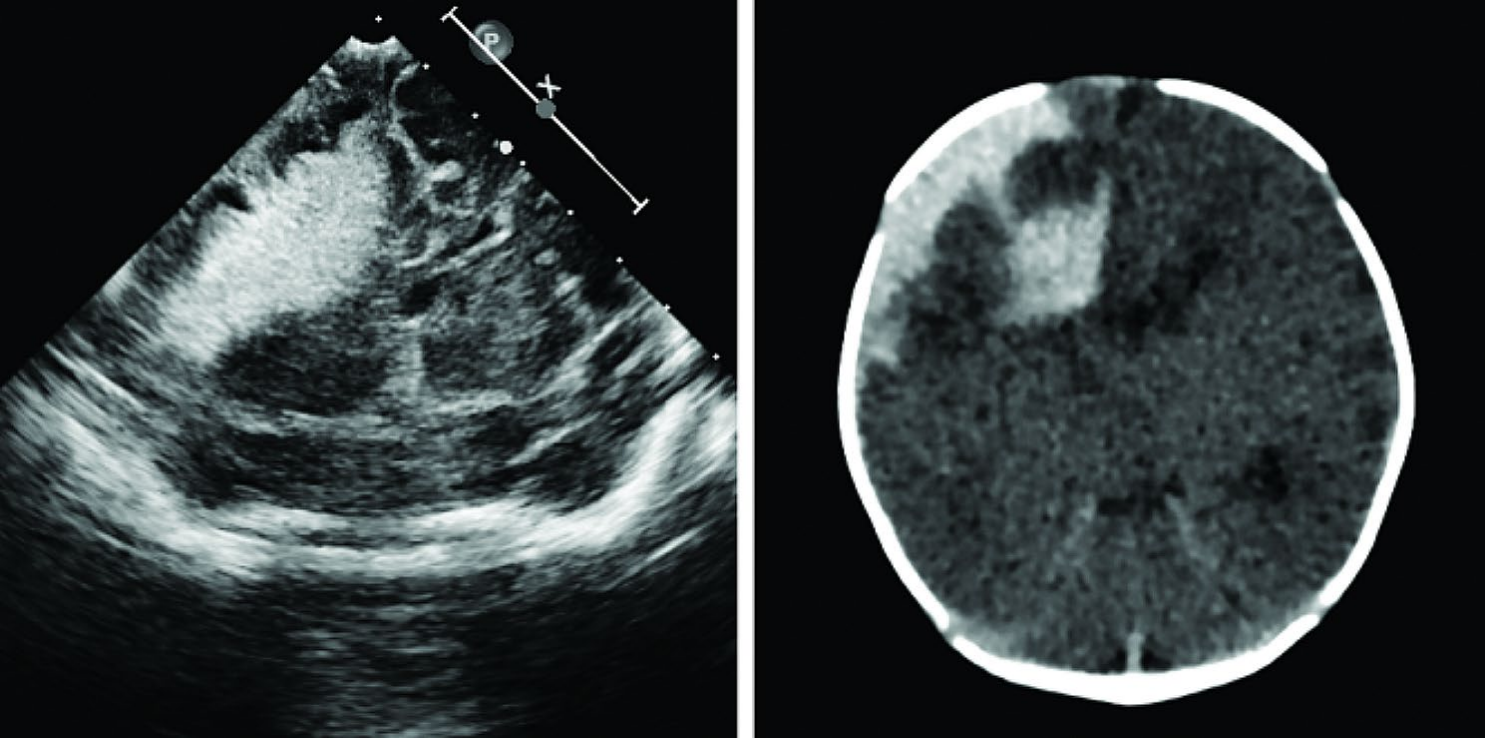
Diagnosis of intracranial hemorrhage. **Left**: Coronal transfontanelle ultrasonographic image showing large hyperechoic mass in the right cerebral hemisphere causing midline shift. **Right**: Coronal noncontrast CT image showing right intraparenchymal hemorrhage with surrounding edema and midline shift. There is also right subarachnoid hemorrhage

**Fig. 3:**
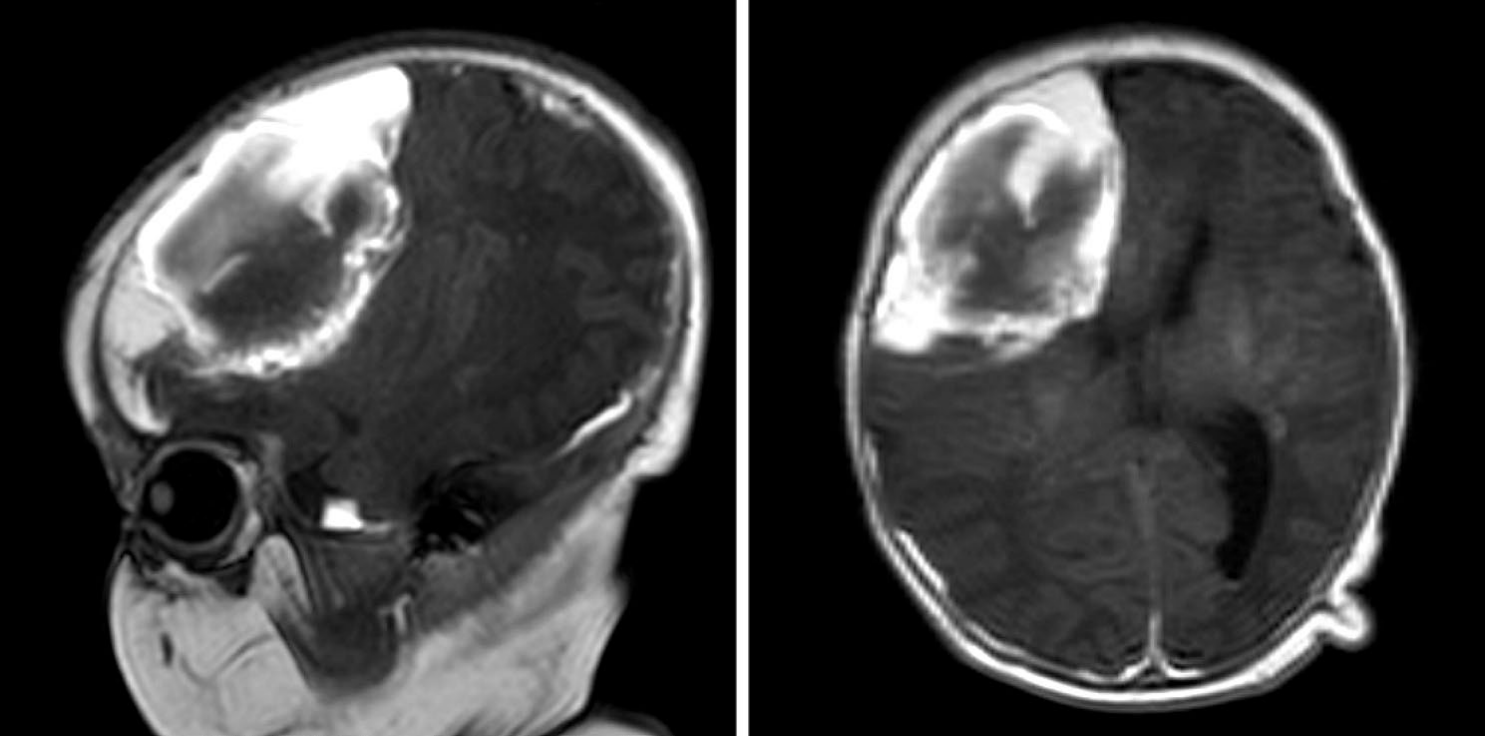
Utility of MRI. Sagittal (**Left**) and axial (**Right**) T1-weighted MR images showing a large intraparenchymal hemorrhage

**Fig. 4:**
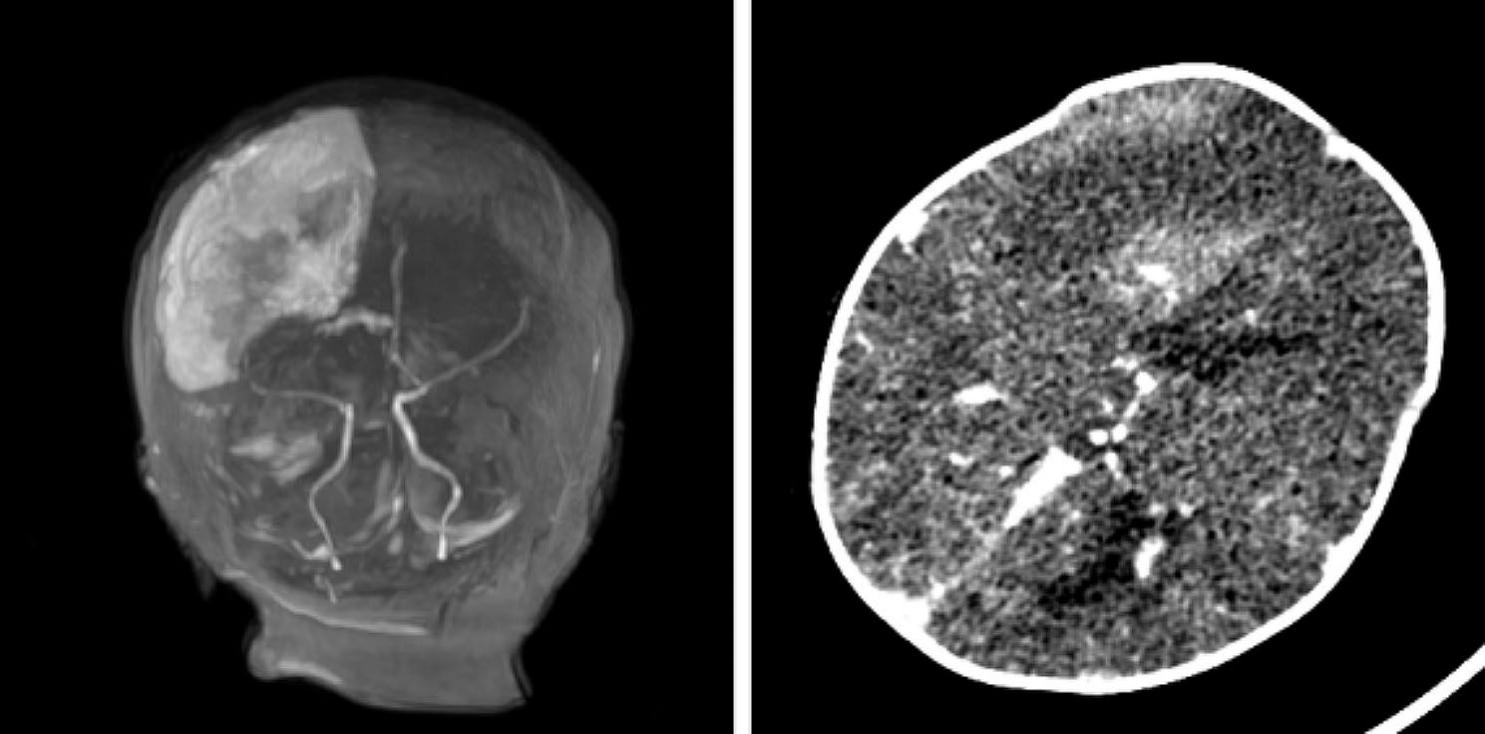
Utility of MRA + MRV and contrast-enhanced CT. TOF MR (**Left**) angiogram showing poor visualization of the right anterior cerebral artery branches. Contrast-enhanced CT image (**Right**) showing a vividly enhancing oval mass located directly adjacent to the parenchymal hemorrhage

The initial cranial examination of the child after admission was ultrasound (Fig. 2 left) followed by computed tomography (CT) (Fig. 2 right), which showed extensive right-sided parenchymal hemorrhage. Magnetic resonance angiography (MRA) and magnetic resonance venography (MRV) showed hemorrhage possibly originating from a malformation of an intracranial artery (Figs. 3 and 4 left). Enhanced CT showed a vividly enhancing lamellar hyperdense shadow with tortuous thickened vascular shadows, consistent with the diagnosis of vascular malformation (Fig. 4 right). Abdominal symptoms (bloody stools and abdominal distension) of NEC started on Day 15 after formula feeding. We searched for cases from 1991 to the present and found no reports of NEC secondary to parenchymal brain hemorrhage in full-term newborns. However, one study noted that NEC is a risk factor for the progression of intraventricular hemorrhage in preterm infants less than 1,000 g, especially if treated surgically [10]. The possible mechanism proposed by the authors is that the inflammation triggered by NEC can lead to the release of pro-inflammatory cytokines and other mediators into the bloodstream. These inflammatory molecules can cross the blood-brain barrier, which is also immature in preterm infants, and induce inflammation within the brain parenchyma. This finding fits with the theory of the gut-brain axis.

In reviewing the management of this case, the diagnosis of intracranial hemorrhage led clinicians to overlook the possibility of abdominal disease in the child. For six days prior to being diagnosed with NEC, the child exhibited intermittent symptoms of poor mental performance, lethargy, and refusal to eat. Initially, these nonspecific symptoms were attributed to the progression of intracranial hemorrhage. The cranial MRI results alleviated our concerns, and the negative result from the abdominal ultrasound on the same day further eased our vigilance toward possible abdominal complications. It wasn’t until the child developed overt clinical symptoms such as abdominal distension and bloody stools that we realized the underlying cause of these symptoms did not originate in the brain but in the gut. This oversight is precisely what motivated us to write this article and underscores a challenge faced by clinicians: the absence of specific indicators for early diagnosis of NEC.

The main identifiable risk factors for NEC in our patient were formula feeding, antibiotic therapy, and ruptured intracranial vascular malformation with hemorrhage. We have attempted to provide the following explanations for the mechanism of NEC after spontaneous parenchymal hemorrhage: On the one hand, the large volume of intracranial hemorrhage, the rapid decrease in hemoglobin, and the subsequent dehydrating cranial pressure-lowering therapy caused inadequate intestinal perfusion; on the other hand, antibiotic therapy has been suggested to increase the risk of NEC in preterm infants by reducing the diversity of the microbiota [11]. However, more proof is needed because this case involves a full-term infant. Additionally, research has indicated that breastfed infants are significantly associated with a reduction in the emergence of NEC because formula-fed and breastfed newborns have different intestinal microbiota compositions [11]. Finally, there is a close connection between the brain and the digestive system. The gut-brain axis is a bi-directional communication pathway linking the gastrointestinal system to the central nervous system [12]. This communication primarily occurs through inflammatory mediators, neural pathways, endocrine signals, and gut microbiota [13]. The gut microbiota largely regulates the components of the gut-brain axis [14]. They can activate neural pathways in the brain, stimulate mucosal immune responses, and influence the brain directly by producing metabolites [15]. Research indicates that the gut microbiota-brain axis is associated with neurodevelopmental defects in preterm infants with NEC [7], and fecal microbiota transplantation suppresses neuroinflammation in mice with traumatic brain injury by modulating peripheral immune cells [16]. Conversely, this bidirectional communication system allows the brain to influence the gut. The pioneering research by Soviet scientist Ivan Pavlov first demonstrated the direct influence of the brain on gastrointestinal function [17]. Norin’s research suggests that head trauma may lead to dysbiosis and disruption of the microbiota in the brain and body, resulting in the production of harmful metabolites and local damage [18]. Houlden’s study found that brain injury induces specific changes in the cecal microbiota by altering autonomic nerve activity and mucin production in mice [19]. After intracerebral hemorrhage, there are alterations in the composition of gut microbiota, accompanied by impaired intestinal barrier function [20, 21]. Therefore, we hypothesize that intracranial hemorrhage may increase the risk of NEC in infants by affecting the communication of gut microbiota. In addition to the influence of the gut microbiota, the stress response induced by cerebral hemorrhage and the release of pro-inflammatory cytokines can activate the hypothalamic-pituitary-adrenal (HPA) axis. Activation of the HPA axis induces cortisol secretion [22]. During this fragile period in early life, the interaction between the stress system and the gut microbiota is altered following intracerebral hemorrhage, leading to dysfunction in several intertwined components of the gut-brain axis and increasing susceptibility to NEC.

Our case suggests that a high level of awareness of the development of NEC should be maintained in the presence of severe intracranial hemorrhage. Severe intracranial hemorrhage in neonates may be a risk factor for the development of NEC. However, its specific pathomechanism needs further study. Ultimately, we hope that through this case, clinicians will strengthen their understanding of the possibility of secondary NEC in neonates with parenchymal hemorrhage to achieve prevention, early detection, and early treatment and minimize functional damage.

## Data Availability

The datasets used and/or analyzed during the current study are available from the corresponding author on reasonable request.

## Abbreviations

HPA: Hypothalamic-pituitary-adrenal
MRA: Magnetic resonance angiography
MRI: Magnetic resonance imaging
MRV: Magnetic resonance venography
NEC: Necrotizing enterocolitis
NICUs: Neonatal intensive care units

## References

[1] Bubberman JM, van Zoonen A, Bruggink JLM, van der Heide M, Berger RMF, Bos AF (2019). Necrotizing enterocolitis Associated with congenital heart disease: a different entity?. J Pediatr Surg.

[2] Stoll BJ, Hansen N, Bell EF, Shankaran S, Laptook AR, Wals h MC (2010). Neonatal outcomes of extremely preterm infants from the NICHD Neonatal Research Network. Pediatrics.

[3] Ostlie DJ, Spilde TL, St Peter SD, Sexton N, Miller KA, Sharp RJ (2003). Necrotizing enterocolitis in full-term infants. J Pediatr Surg.

[4] Maayan-Metzger A, Itzchak A, Mazkereth R, Kuint J (2004). Necrotizing enterocolitis in fullterm infants: case–control study and review of the literature. J Perinatol.

[5] Short SS, Papillon S, Berel D, Ford HR, Frykman PK, Kawaguchi A (2014). Late onset of necrotizing enterocolitis in the full-term infant is associated with increased mortality: results from a two-center analysis. J Pediatr Surg.

[6] Lin PW, Stoll BJ (2006). Necrotising Enterocolitis Lancet.

[7] Wang Y, Hang C, Hu J, Li C, Zhan C, Pan J (2023). Role of gut-brain axis in neurodevelopmental impairment of necrotizing enterocolitis. Front Neurosci.

[8] Bellodas Sanchez J, Kadrofske M (2019). Necrotizing Enterocolitis Neurogastroenterol Motil.

[9] Mohotti JE, Carter NS, Zhang VJW, Lai LT, Xenos C, Asadi H et al. Neonatal intracranial aneurysms: case report and review of the literature. J Neurosurg Pediatr. 2018;21(5):471–7. 10.3171/2017.10.PEDS17226 29498602

[10] Jen HC, Graber JJ, Hill JL, Alaish SM, Voigt RW, Strauch ED. Surgical necrotizing enterocolitis and intraventricular hemorrhage in premature infants below 1000 g. J Pediatr Surg. 2006;41(8): 1425–30. 10.1016/j.jpedsurg.2006.04.019 16863849

[11] Pammi M, Cope J, Tarr PI, Warner BB, Morrow AL, Mai V et al. Intestinal dysbiosis in preterm infants preceding necrotizing enterocolitis: a systematic review and meta-analysis. Microbiome. 2017;5(1):31. 10.1186/s40168-017-0248-8 PMC5343300 28274256

[12] Mui NW, Uddin A, Fortunato MP, Nolan BE, Clare KM, Lui AK et al. The gut-brain connection: inflammatory bowel disease increases risk of acute ischemic stroke. Interv Neuroradiol. 2023;0:0. 10.1177/15910199231170679 PMC12202956 37157802

[13] Weaver JL (2021). The brain-gut axis: a prime therapeutic target in traumatic brain injury. Brain Res.

[14] Collins SM, Surette M, Bercik P (2012). The interplay between the intestinal microbiota and the brain. Nat Rev Microbiol.

[15] Reutov VP, Sorokina EG (2022). Causal relationship between physiological and pathological processes in the brain and in the gastrointestinal tract: the brain-intestine Axis. Biophys (Oxf).

[16] Hu X, Jin H, Yuan S, Ye T, Chen Z, Kong Y (2023). Fecal microbiota transplantation inhibited neuroinflammation of traumatic brain injury in mice via regulating the gut-brain axis. Front Cell Infect Microbiol.

[17] Tansey EM (2006). Pavlov at home and abroad: his role in international physiology. Auton Neurosci.

[18] Norins LC (2019). The beehive theory: role of microorganisms in late sequelae of traumatic brain injury and chronic traumatic encephalopathy. Med Hypotheses.

[19] Houlden A, Goldrick M, Brough D, Vizi ES, Lénárt N, Martinecz B (2016). Brain injury induces specific changes in the caecal microbiota of mice via altered autonomic activity and mucoprotein production. Brain Behav Immun.

[20] Li W, Wu LX, Huang BS, Yang LJ, Huang JQ, Li ZS (2022). A pilot study: gut microbiota, metabolism and inflammation in hypertensive intracerebral haemorrhage. J Appl Microbiol.

[21] Ferguson JF, Aden LA, Barbaro NR, Van Beusecum JP, Xiao L, Simmons AJ (2019). High dietary salt-induced dendritic cell activation underlies microbial dysbiosis-associated hypertension. JCI Insight.

[22] de Punder K, Pruimboom L (2015). Stress induces endotoxemia and low-grade inflammation by increasing barrier permeability. Front Immunol.

